# Acute Limb Ischemia in the Young: Clinical Insights From a Primary Aortic Myxoma

**DOI:** 10.1016/j.jaccas.2026.110411

**Published:** 2026-10-06

**Authors:** Shingo Kunioka, Shogo Takahashi, Hiroyuki Kamiya

**Affiliations:** Department of Cardiac Surgery, Asahikawa Medical University, Asahikawa, Japan

**Keywords:** aorta, cardiovascular disease, circulation, echocardiography, thoracotomy, thrombus, vascular disease

**Figure:**
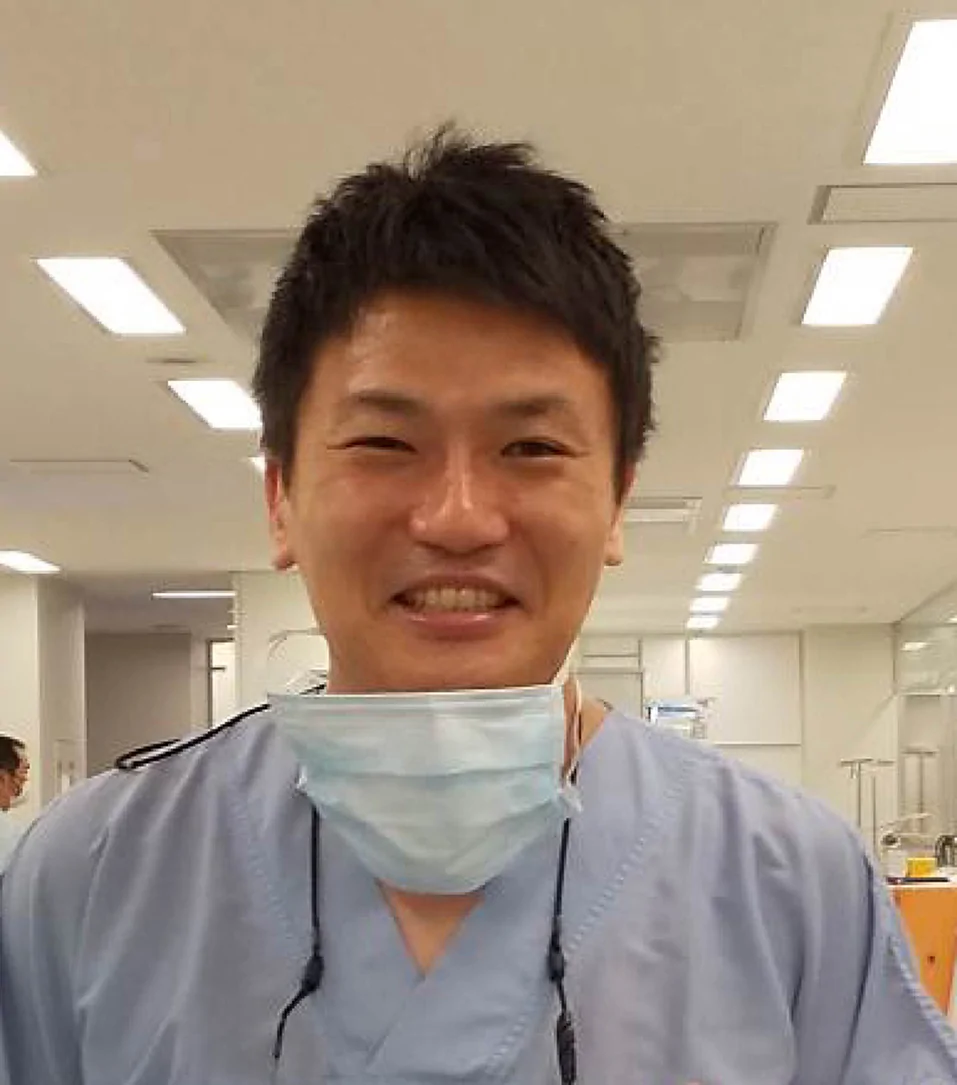

## Diagnostic Challenges in Unexplained Systemic Embolism

Acute limb ischemia is a vascular emergency in which rapid restoration of blood flow must be balanced against identification and control of the underlying source. In situ thrombosis and arterial embolism remain the principal mechanisms, and atrial fibrillation is a frequent cause of peripheral arterial embolization in older patients. According to previous reports of peripheral arterial embolism, two-thirds of patients were in atrial fibrillation at presentation.^1^^,^^2^ However, the diagnostic approach must change substantially when acute limb ischemia occurs in a young patient without established lower-extremity arterial disease, atrial fibrillation, structural heart disease, or another conventional embolic substrate.

In this issue of *JACC: Case Reports*, Salazar Ulacia et al^3^ demonstrate this concept in a particularly compelling manner. A 38-year-old patient presented with acute upper- and lower-extremity ischemia and experienced immediate recurrent embolization during peripheral thromboembolectomy. Further evaluation revealed a mobile ascending aortic lesion—subsequently confirmed as a myxoma—along with a second lesion in the descending thoracic aorta treated with thoracic endovascular aortic repair. This rare presentation offers crucial clinical insights into evaluating acute limb ischemia in younger adults, recognizing the imaging limits of mobile intra-aortic masses, strategizing cerebral protection during source control, and addressing the oncological uncertainty of endovascularly excluding a potential tumor.

## When the Embolic Source Lies Within the Aorta: Imaging Has Limits

Computed tomographic angiography (CTA) is central to evaluating acute limb ischemia, rapidly defining arterial occlusion and extending the search proximally into the thoracic and abdominal aorta. Current guidelines support urgent imaging provided it does not delay revascularization of a threatened limb.^1^ However, CTA captures morphology only at a single moment. Although it readily identifies intraluminal filling defects, distinguishing thrombus from tumor, vegetation, or atypical dissection remains challenging—especially for small, noncalcified, poorly enhancing, or thrombus-covered lesions. Electrocardiographically-gated CTA mitigates motion artifacts in the ascending aorta. Indeed, electrocardiographically-gated imaging recently identified a thin stalk and marked mobility in an ascending aortic mural thrombus that nongated computed tomography had mischaracterized as a stable lesion, substantially altering the operative strategy.^4^ Transesophageal echocardiography (TEE) offers complementary real-time information about mobility, attachment, and hemodynamic behavior, but it is not infallible. The distal ascending aorta and proximal arch constitute a recognized “blind spot” because the air-filled trachea and main bronchi intervene between the esophagus and aorta.^5^ A previous study also showed that small, localized proximal dissections may be missed because of interference from tracheobronchial air.^6^ Thus, although TEE remains a valuable diagnostic tool, imaging artifacts remain a significant limitation.

The converse problem is equally important: ultrasound artifacts may imitate an intimal flap and produce a false-positive diagnosis of aortic dissection. Evangelista et al^7^ found 93 artifacts in 56 of 101 patients without ascending aortic dissection.

In the present case of Salazar Ulacia et al,^3^ intraoperative TEE raised concern for Stanford type A acute aortic dissection. Surgical exploration under circulatory arrest instead revealed a tumor-like intraluminal lesion, later confirmed to be a myxoma.^3^ This case does not reflect a failure of TEE, but underscores that although imaging delineates mass morphology and embolic risk, tissue diagnosis requires histopathology. When CTA and TEE findings diverge, a broad differential—including thrombus, dissection, and benign or malignant aortic neoplasms—must be maintained.

## Cerebral Protection and Source Control

Once a highly mobile lesion in the ascending aorta is identified, establishing cardiopulmonary bypass can itself create an embolic hazard due to arterial cannulation and aortic manipulation before cerebral isolation. Salazar Ulacia et al^3^ selected brachiocephalic arterial inflow and unilateral selective cerebral perfusion, which enabled the initiation of cardiopulmonary bypass while preventing stroke and allowing the ascending aorta to be opened during circulatory arrest. Similar to a related report using bilateral axillary cannulation, temporary left common carotid artery occlusion during cardiopulmonary bypass initiation, and unilateral selective cerebral perfusion during circulatory arrest, this approach represents an effective perfusion strategy for patients with an embolic source in the ascending aorta.^4^

The decision to perform initial thromboembolectomy was clinically sound. By referral, 12 hours had elapsed, and the patient exhibited lower-extremity sensorimotor deficits—a clear sign of an immediately threatened limb. Delaying revascularization risked irreversible neuromuscular damage and amputation. Moreover, the intraoperative re-embolization highlights the need to address the embolic source concurrently with limb salvage.

## A Hybrid Strategy to Prevent Further Embolization

The descending thoracic lesion carried a risk of recurrent embolization and required prompt intervention. Descending aortic replacement was considered a more definitive solution; open resection with Dacron graft reconstruction would have provided complete lesion removal, direct pathological evaluation, and definitive aortic-wall reconstruction. However, subjecting the patient to an additional surgical procedure shortly after major cardiac surgery involving circulatory arrest presented a significant physical burden, making this option less attractive. On the other hand, thoracic endovascular aortic repair was expected to prevent embolization by pinning the lesion between the aortic wall and the stent graft, offering a rapid, minimally invasive approach to immediately mitigate embolic risk. However, the key consideration with this technique—beyond requiring meticulous catheter manipulation—is that a lesion strongly suspected to be an endovascular tumor is left between the vessel wall and the stent graft. If the descending lesion was indeed a myxoma, although histologically benign, the possibility of future growth causing recurrent embolization, or even the presence of a distinct malignant aortic neoplasm, cannot be entirely ruled out. When adopting a hybrid strategy, one must carefully weigh the benefit of minimal invasiveness against the disadvantage of leaving a neoplastic lesion within the vessel, making the long-term follow-up results of this case report particularly intriguing.

## Conclusions

This case highlights that acute or recurrent limb ischemia in young patients requires an active search for unusual proximal embolic sources, such as aortic tumors. When mobile ascending aortic lesions are present, perfusion planning must strictly prioritize embolic protection prior to aortic manipulation. A hybrid strategy combining open surgical excision with endovascular exclusion offers rapid, effective source control in high-risk emergencies when paired with vigilant long-term surveillance.

## Funding Support and Author Disclosures

The authors have reported that they have no relationships relevant to the contents of this paper to disclose.
